# Petri Nets with Fuzzy Logic (PNFL): Reverse Engineering and Parametrization

**DOI:** 10.1371/journal.pone.0012807

**Published:** 2010-09-20

**Authors:** Robert Küffner, Tobias Petri, Lukas Windhager, Ralf Zimmer

**Affiliations:** Institut für Informatik, Ludwig-Maximilians-Universität, München, Germany; Center for Genomic Regulation, Spain

## Abstract

**Background:**

The recent DREAM4 blind assessment provided a particularly realistic and challenging setting for network reverse engineering methods. The *in silico* part of DREAM4 solicited the inference of cycle-rich gene regulatory networks from heterogeneous, noisy expression data including time courses as well as knockout, knockdown and multifactorial perturbations.

**Methodology and Principal Findings:**

We inferred and parametrized simulation models based on Petri Nets with Fuzzy Logic (PNFL). This completely automated approach correctly reconstructed networks with cycles as well as oscillating network motifs. PNFL was evaluated as the best performer on DREAM4 *in silico* networks of size 10 with an area under the precision-recall curve (AUPR) of 81%. Besides topology, we inferred a range of additional mechanistic details with good reliability, e.g. distinguishing activation from inhibition as well as dependent from independent regulation. Our models also performed well on new experimental conditions such as double knockout mutations that were not included in the provided datasets.

**Conclusions:**

The inference of biological networks substantially benefits from methods that are expressive enough to deal with diverse datasets in a unified way. At the same time, overly complex approaches could generate multiple different models that explain the data equally well. PNFL appears to strike the balance between expressive power and complexity. This also applies to the intuitive representation of PNFL models combining a straightforward graphical notation with colloquial fuzzy parameters.

## Introduction

The inference of biological networks based on gene expression measurements is a complex task. A range of approaches have been developed for that purpose, which is in turn reflected by a range of corresponding reviews [1]–[7]. Basic principles to derive relationships between genes or proteins include ordinary differential equations (ODE) [8]–[10], mutual information [11] and Bayesian networks [12].

Predictions from the available methods are currently quite unreliable as shown in several comparative studies on *in silico* networks [13]–[16]. For instance, precisions of less than 30% have been observed in [14] for all approaches investigated. This might be due to the fact that most methods were developed to exploit either (static) interventional datasets such as knockout experiments or dynamic datasets such as time courses, but not both [4].

Whether the incorporation of a broad range of datasets can increase the reliability of network reconstruction is explored by the DREAM competitions that conduct blind assessments of network reverse-engineering approaches [17]. The *in silico* part of DREAM4 (2009) provided time course datasets together with complex knockout, knockdown and multifactorial perturbation datasets.

We present a network inference approach based on Petri Nets with Fuzzy Logic (PNFL) [18]. Similar to ODEs but in contrast to Bayesian or mutual information networks, PNFL enables a simulation of the models. In contrast to the more detailed ODEs, PNFL employs a simpler rule based discrete modeling system. The simulation is important for the investigation and refinement of mechanistic network models in order to capture the dynamic behavior of systems in addition to their topology. In case of DREAM4, we simulate to re-generate the provided datasets. The objective of our inference approach is the reconstruction of models by optimizing the agreement between all of the datasets provided in the challenge and those generated by PNFL. Heterogeneous datasets can thus be exploited and scored in a unified way.

In the following, we briefly summarize the DREAM4 setting, introduce Petri Nets and PNFL and outline our approach to simulate and reconstruct PNFL models. Subsequently, we describe the results we obtained in the DREAM4 *in silico* size ten challenge.

## Methods

### Setting of the DREAM4 *in silico* size ten challenge

#### Problem statement

The *in silico* part of DREAM4 aims at the reconstruction of gene regulatory networks where effects are propagated via directed transcription factor (TF, i.e. the effector protein) → target gene relationships. TFs are synthesized from their corresponding genes and can thus be themselves the targets of other TFs. Other kinds of relationships (e.g. alternative splicing, protein modification, transport, metabolic reactions) were not considered.

The task is the automated reverse engineering of the directed topology of five different networks with ten nodes per network. The topology to be predicted merges genes and their products (i.e. the TFs) into single nodes. All networks contain cycles, but no self loops. No direct information on the edges is given. Instead, networks are to be inferred from the provided gene expression datasets (see below) alone. In a bonus round, participants used their reconstructed networks to simulate dual knockout perturbations. The problem statement, evaluation and datasets are described in more detail on the DREAM website (http://wiki.c2b2.columbia.edu/dream/index.php/D4c2).

#### Evaluation

After the challenge, submissions were evaluated against the true topology based on the area under the precision-recall curve (AUPR) and the area under the receiver-operator characteristics curve (AUROC). We will focus our discussion on the AUPR. Roughly speaking, an AUPR of 50% means that for each correctly predicted edge an erroneous edge is predicted as well. The sign of the edges (activation vs. inhibition) is not considered in the DREAM4 evaluation. Dual knockout predictions were compared against the true equilibrium values via the mean squared error (MSE). The evaluation is described in more detail in [19].

#### Gene expression datasets

The approach for dataset generation was developed by Marbach et al. [20], [17]. Five time course (TC) datasets were provided. At the beginning of the TC, strong perturbations were applied to the basal transcription levels of about a third of all genes. Halfway through the TC the perturbation was removed so that the network relaxed to the wild type (WT) equilibrium state (5 TC * 20 measurements * 10 genes = 1000 values). All other datasets contained equilibrium gene levels only. Ten single gene knockout (KO), knockdown (KD) and multifactorial (MF) perturbations (3 * 10 perturbations * 10 genes = 300 values) were provided. Compared to the wild type (WT), basal transcription levels of KO and KD target genes were reduced to 0% and 50%, respectively. MF datasets were generated by applying moderate perturbations to the basal activation levels of all genes in the network. Thus, MF datasets could be regarded as transcriptional variations between different individuals. Given gene levels were scaled to be in the range [0, 1].

### Petri Nets

The application of Petri net theory for modeling and analysis of biological networks is well established in the field of systems biology [21]–[24]. Petri nets are graph representations of networks consisting of two types of nodes: *places*, representing entities like proteins, genes, metabolites etc, and *transitions*, representing reactions or, in general, state changes of entities. The state of an entity is defined by the *tokens* that represent the *marking* of the according place and the overall system state by the marking of the Petri net. Directed edges (*arcs*) connect places to transitions (input arcs) or transitions to places (output arcs). These arcs not only depict which entities influence reactions or are influenced by them, but they also exactly define the effects of a reaction, e.g. by specifying the amount of substrate consumed and the amount of product produced during a reaction (the *firing* of a transition). For a detailed description of classical Petri nets see [25]. In addition, there exists a wide variety of extensions of Petri nets [26]. A Petri Net with Fuzzy Logic (PNFL) can be defined as an instance of a hybrid functional Petri net (HFPN) [27].

**Figure 1:**
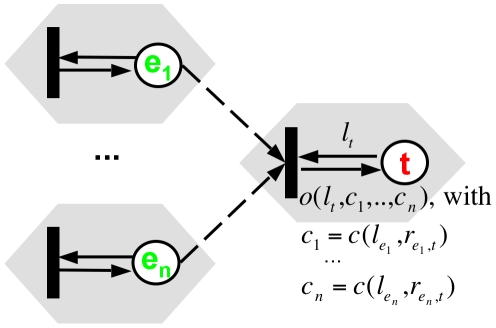
Petri Nets with Fuzzy Logic (PNFL). In Petri nets, states such as effector (e) or target (t) gene levels are represented by places and are depicted as circles. State changes are represented by transitions and are depicted as boxes. Effect arcs (i.e. effector place-transition arcs) define the effectors influencing a target gene via the transition. Firing transitions leaves the marking of the effector places unchanged (test arcs, dashed). After the application of rule tables *r_e,t_* to effector gene levels *l_e_* (function *c*, eq. 1–3), the target gene levels *l_t_* are updated by the output function *o* (eq. 4–6). In Fig. 6, Fig. 8 and Fig. 10, we represent a transition and its output place as a simplified hexagonal node. The reconstruction determines the topology ( = effect arcs) and the parametrization ( = rule tables and combination operators) of PNFL models.

In PNFL models of gene regulatory networks, the activity of a target gene *t* is controlled by a single transition that discharges into a single output place (see Fig. 1). The marking of this place represents *t*'s numerical gene level *l_t_*. The relationship between an effector place and a target place is called an *effect*. It is mediated by an *effect arc* connecting an effector-gene place to the transition that controls the target place.

Transitions are always enabled as each place always contains a valid (real-valued) token. Firing a transition removes the old marking on the target place via a target place-transition arc (Fig. 1). After quantifying the effect strength based on the effector gene level (via function *c*, see eq. 1–3), a new marking is assigned to the target place by the output function *o* (eq. 4–6). The marking on effector places remains unchanged (test arcs).

A transition, its output place and their connecting arcs can be replaced by hexagonal nodes (Fig. 1) to simplify the representation. In this reduced form, only hexagons and their connecting effect arcs remain as all transitions and places are replaced. Effect arcs will be attached to or detached from transitions during the network reconstruction process (section Reconstruction) thus connecting different hexagonal nodes in the reduced Petri net representation.

### Modeling of gene regulatory relationships with PNFL

The evaluation of effects using fuzzy logic involves a three-step procedure that consists of fuzzification, the application of effector rules and defuzzification.

#### Fuzzification

**Figure 2:**
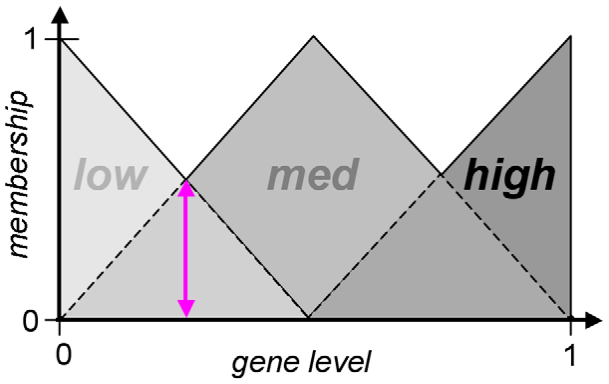
Fuzzification and defuzzification. We use triangular membership functions to fuzzify the continuous gene levels of an effector *e* into fuzzy sets. As shown by the magenta arrow, a continuous gene level of *l_e_* = 0.25 is fuzzified into the fuzzy value <*L*(*low,l_e_*) = 0.5, *L(med,l_e_)* = 0.5, *L*(*high,l_e_*) = 0.0>. This can be reversed by defuzzification without loss of information.

In a first step, the continuous gene level *l_e_* ∈ [0,1] of an effector *e* is transformed (fuzzified) into the fuzzy value <*L*(*low,l_e_*), *L*(*med*,*l_e_*), *L*(*high*,*l_e_*)> by triangular membership functions (eq. 1, Fig. 2):
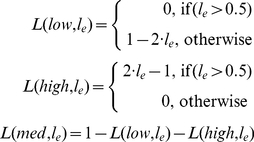
(1)Such membership functions are called *fuzzy sets* [28]. Contrary to a classical set, where an object is either contained in the set or not (two-valued logic, {0,1}), a fuzzy set assigns a degree of membership from the interval [0,1] to each object. Thus, the fuzzy value resulting from the fuzzification of a gene level *l_e_* with respect to three fuzzy sets *S*∈{*low*, *med*, *high*} can be interpreted as a fuzzy discretization.

#### Application of effector rules

**Figure 3:**
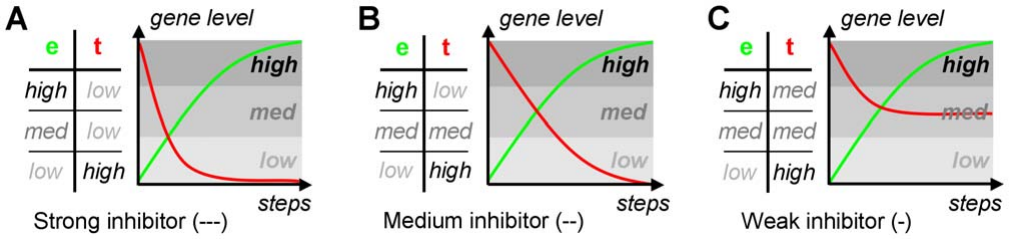
Rule tables. Given fuzzy effector gene levels, we describe the behavior of the targets by rule tables. Rule tables define sign and strength of effects. Fully active strong (−−−, A) or medium (−−, B) inhibitors result in low target activity, which is in contrast to weak inhibitors (−, C). The corresponding strong (+++), medium (++) and weak (+) activator rule tables are constructed by exchanging *high* by *low* and *low* by *high* in the target column.

Based on the discretization defined by fuzzy sets, the properties of regulatory relationships are modeled by rule tables *r_e,t_* (Fig. 3) in analogy to Boolean network models. Rule tables (as used in DREAM4) define three levels of effect strength for both activation (+++, ++, +) and inhibition (−−−, −−, −). A rule table *r_e,t_*:*S*→*S* maps each effector set *E*∈*S* to a corresponding target set *T*∈*S*. The application of a rule by the sum-product logic [29] results in a fuzzy rule consequent *C*:
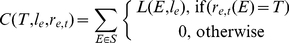
(2)Applying eq. 2 to all sets *T*∈*S* results in a fuzzy value <*C*(*low,l_e_,r_e,t_*), *C*(*med,l_e_,r_e,t_*), *C*(*high, l_e_,r_e,t_*)> describing *e*'s effect on the target gene *t*, i.e. it is a fuzzy discretization of the proposed effect.

#### Defuzzification

**Figure 4:**
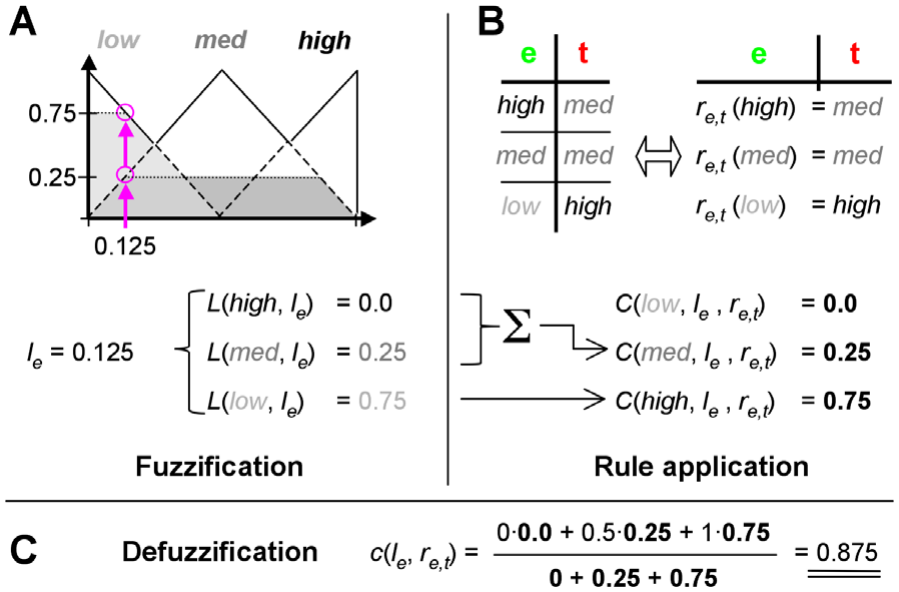
Fuzzy effect calculation example. In this example, the gene level of effector *e* is *l_e_* = 0.125. It is transformed (fuzzified, panel A) into the fuzzy gene level *L* by application of eq. 1. In panel B, the rule table *r_e,t_* (Fig. 3C) is applied to describe the influence of *e* onto its target gene *t* by the rule consequent *C*. *C* is derived by eq. 2, yielding the fuzzy value <0, 0.25, 0.75> (panel B). The real valued influence of *e* onto *t*, *c*(*l_e_, r_e,t_*) = 0.875, is calculated by defuzzification (panel C). Such a calculation is performed for all effectors of the target gene *t* individually. The influences are combined by eq. 4 or eq. 6 (not shown here, see text).

By center of gravity defuzzification we obtain a continuous rule consequent *c*:
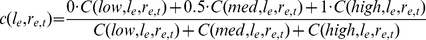
(3)with the centers of gravity at 0, 0.5 and 1. Note that due to our choice of fuzzy sets and rule tables the value of the denominator always equals to one. An example calculation involving eq. 1–3 is shown in Fig. 4.

#### Combination of effects

**Figure 5:**
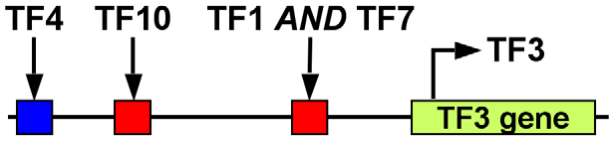
Combinatorial gene regulation. The regulatory logic of different transcription factors (TFs) regulating a target gene used in DREAM4 was disclosed after the challenge. TFs are assumed to bind to cis-regulatory modules (CRMs) to regulate the expression of target genes. Individual CRMs act as enhancers (red) or repressors (blue) of gene regulation. The bound states of different CRMs (e.g. by TFs 4 and 10) are mutually *independent*. A complex of TFs regulating a given CRM can be represented as *AND* operator. TFs 1 and 7 are mutually *dependent* to form the complex and regulate the gene. In turn, a complex of TFs controlling a repressing CRM can be implemented by the *OR* operator (not shown). The effects of several CRMs on the activity of the target gene are averaged (*MEAN* operator). In contrast to the arbitrary combination of operators in the DREAM4 approach, PNFL selects only a single operator (*AND*, *OR* or *MEAN*) per target gene (see Methods and Results). The depicted regulation of gene 3 was taken from network 5 (see Fig. 10A).

If several effectors regulate a target gene, their combined effect on the target can be modeled by logical operations [30]. We model two kinds of dependent regulation by the minimum of the effects (*AND* operator) or the maximum of the effects (*OR* operator). The average (*MEAN*) models the independent regulation of a target by its effectors. Dependent and independent regulation are described in Fig. 5. The combination logic currently used in PNFL allows only a single operator (either *AND*, *OR* or *MEAN*) to be selected per target gene regardless of the number of effectors, see eq. 4 and eq. 6.

### PNFL simulation

Before simulation, gene levels are initialized to their wild type levels as provided by DREAM4. Let *t* be a gene targeted by *n* effectors *e_1_*,…,*e_n_*. In each simulation step, updates *u* of the levels of all genes are computed from the continuous rule consequents 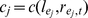:
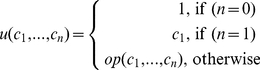
(4)with *op* ∈ {*AND*, *OR*, *MEAN*}. Subsequently, *u* is applied to the gene levels *l_t_* of all target genes via the output function *o* (eq. 5) at once.
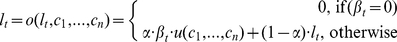
(5)The scaling parameter *α* (Table 1) aligns the PNFL generated time courses to the provided time courses. The transcription rate parameter *β_t_* tunes the transcription rate of gene *t*, with *β_t_* = 1 for the wild type transcription rate.

**Table 1:** Parameters used for PNFL based reconstruction.

Parameter descriptions	Equation	Values/*Lists of values* a
Rule tables *r*: effect strength	2	(*M*), (*W*, *S*), (*W*, *M*, *S*)^b^
Combination operators	4, 6	(*MEAN*), (*OR*, *AND*), (*OR*, *AND*, *MEAN*)
Update ratio *α*	5	*0.4*, *0.5*, *0.6*
Regularization parameter *reg*	7	*0.005*, *0.002*, *0.001*, *0.0005*, *0.0002*
Weight time course *w_TC_*	7	1
Weight knockout *w_KO_*	7	8
Weight knockdown *w_KD_*	7	6
Weight multifactorial *w_MF_*	7	4
Simulated annealing parameter *k*	8	0.02

^**a**^Parameters from *lists* are randomly selected for ensemble predictions (see Submission).

^**b**^Degrees of effect strength, W = weak = (+,−), M = medium = (++,−−) and S = strong = (+++,−−−).

#### Knockout, knockdown and double knockout data

Gene perturbations are simulated by reducing the transcription rate *β_t_*. In case of a knockout or knockdown simulation, *β_t_* of the perturbed gene *t* is set to 0 or 0.5, respectively. Similarly, double knockout simulations can be performed.

#### Time course data

Time course datasets were provided by DREAM4 to show the impact of strong gene perturbations (about a third of all genes) on a network as well as the relaxation to the wild type equilibrium state after removing the perturbations.

A perturbation is represented as an additional (hidden, i.e. unobserved) node in the network. During reconstruction, we infer perturbation targets together with effector targets, as both were not disclosed in the challenge. Initially, we use eq. 6 instead of eq. 4 for all genes *t* directly affected by the perturbation. For a time course *i*, eq. 6 includes the perturbation term 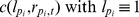, where *p_i_* is an additional perturbation effector with corresponding rules 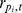. The perturbation is disabled halfway through the time course via switching back to eq. 4 thereby allowing the network to return to its wild type state. The reconstructed networks thus consist of 15 variables: 10 genes and 5 perturbation variables for the 5 different time courses.
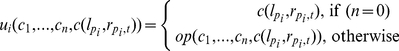
(6)


#### Multifactorial data

DREAM4 also provided equilibrium values for multifactorial (MF) perturbations. Here, the basal transcription levels of all genes in the network were perturbed, but to a lesser degree compared to the time course perturbations. In contrast to the time courses, we do not compute additional rule consequents for the MF data. Instead, we test how well MF target gene levels can be generated if the PNFL wild type rules are applied to the provided MF effector gene levels. The MF target gene levels are thus approximated, as the basal activation changes are not reflected in the PNFL rules.

Differences between the PNFL and DREAM MF gene levels can be due to three reasons: (1) the inferred effects or their parametrization are inadequate: this should be corrected by the reconstruction method, (2) noise and (3) the MF changes to the basal transcription levels. Reasons (1) and (2) apply equally to all of the datasets. For reason (3) we did not account for, so deviations will be somewhat larger than for the other datasets. Therefore, we use lower weights for MF data in the objective function (Table 1).

### Reconstruction

#### Overview

**Figure 6:**
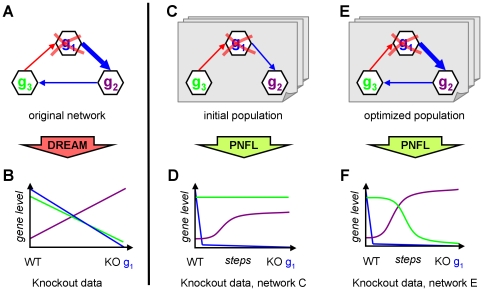
Overview network reconstruction. To reconstruct the original network (A) we mimic the DREAM4 data generation process (A→B). The knockout (KO) of gene 1 is depicted as an example data set in the lower panels. Our reconstruction starts from a randomly initialized population (C) and proceeds through network changing moves. After each move, data is generated by PNFL (D) and compared against the DREAM data (B). We implemented moves on single networks in the population and crossover moves that copy features between pairs of networks. Thereby, favourable features are propagated throughout the population, which eventually leads to improved networks (E) and corresponding datasets (F). Note that - in contrast to the PNFL simulation (D,F) - only equilibrium values were given for knockout experiments in DREAM4 (B). Edges denote effect strength (thickness) and sign (activation = red, inhibition = blue).

We construct PNFL models by inferring and parametrizing relationships between genes via appropriate rule tables. Starting from a randomly initialized PNFL model our reconstruction approach (Fig. 6) proceeds via four steps: (1) The topology and parametrization of the initial network are modified by the application of moves. (2) After each move, data is simulated by PNFL and (3) compared to the original data via an objective function. Finally, (4) we use a simulated annealing protocol to decide if a given move should be accepted or rejected. The network optimization thus targets at the best possible agreement between the DREAM4 provided and PNFL simulated datasets.

Note that the networks discussed here always include 10 genes and the PNFL models always contain one place and one transition for each gene. Topological changes of the PNFL models only involve attachment or detachment of input arcs.

#### Move set and move probabilities

Starting from a population of randomly initialized networks, the reconstruction proceeds one network modifying move at a time. Each move modifies a single target gene. After a move, data is generated by PNFL and compared against the DREAM data (Fig. 6). We implemented moves on individual networks that add or remove effects (i.e. effect arcs), switch the effect combination logic *op *∈ {*AND*, *OR*, *MEAN*} and increase or decrease the effector strength via selecting the corresponding rule tables *r *∈ {+++, ++, +, −, −−, −−−}. Each network in the population evolves both independently by the moves mentioned before but also by a set of crossover moves. The crossover moves copy effect strength, combination logic or effects between two individuals.

During reconstruction, particular moves are selected from the move set with a move probability that is proportional to the past move acceptance probability for that move.

#### Objective function

The quality of the reconstructed networks is evaluated by an objective function *dist*. It is based on the Pearson correlation coefficients *ρ_t_* of the target genes *t* and a regularization term. Lower values of *dist* indicate a better agreement between the DREAM dataset vectors ***x_t_*** and the PNFL dataset vectors ***y_t_*** and thus better PNFL models. The vectors ***x_t_*** and ***y_t_*** are formed by the concatenation of all four kinds of datasets (10 knockout, 10 knockdown, 100 time course, 10 multifactorial values per gene). An additional vector ***w*** = (*w_KO_,…,w_KO_, w_KD_,…,w_KD_, w_TC_,…,w_TC_, w_MF_,…,w_MF_*) weights the data points with dataset specific weights (Table 1). All three vectors are of length 130. The weighted *ρ_t_* is calculated based on the weighted covariance and the weighted mean (not shown). A model coefficient *ρ* is calculated as the average of the gene coefficients *ρ_t_*. In addition, we introduced a regularization parameter *reg*. It allows us to control |*Network*|, i.e. the number of edges ( = effect arcs) in the models.
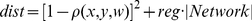
(7)Note that DREAM4 provided only equilibrium values for the knockout, knockdown and multifactorial datasets. Only for the time course datasets gene levels for different measurements were available and are used for the calculation of *dist*.

#### Simulated annealing

We employ simulated annealing to decide if a network changing move is accepted or rejected. That is, we always accept moves that improve the network with respect to the objective function *dist*. We accept inferior networks with a probability *p* calculated from the Boltzmann distribution parametrized by *k* (Table 1). Essentially, moves that only slightly increase *dist* are accepted more frequently, especially if the temperature *T* is high. *T* decreases linearly during the reconstruction run from one to zero.
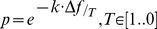
(8)


#### Submission

The DREAM4 submission format required to rank effects by their prediction confidence. We therefore chose a consensus approach to predict an ensemble of networks. Consensus prediction approaches have been successfully applied to network reconstruction before [31]. We carry out 100 reconstruction runs with different parameter settings (Table 1) and random seeds. Ranking is based on the effect prediction confidence calculated as the fraction of networks that included the given effect. This automated approach was the same for each of the submitted nets. No manual processing was performed. For the visualization and description of effects or networks, we assume an effect to be predicted if the prediction confidence is 50% or above.

## Results

### Size of the model space

The number of possible models *m* depends on the number of genes *g* = 10, the number of time courses *tc* = 5, the number of rule tables *r* = 6 (Fig. 3) and the number of combination operators |*op*| = 3 (eq. 4). The number of models *m* depends on the number of time courses *tc* because each time course introduces an additional perturbation variable. As we do not restrict the number of effectors, a gene can be affected by zero, one or up to *n* = *g*−1+*tc* = 14 variables, i.e. 9 other genes (as self interactions were not allowed) and 5 perturbation variables (eq. 6).
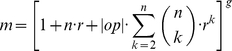
(9)In the given setting, the size of the model space is 1.2*10^123^. Thus, a heuristic search strategy is necessary to detect high scoring networks.

### Reconstruction run time

The most time expensive steps are the simulations needed to calculate the objective function after each move. Each move requires 35 simulations, i.e. 5 time courses, 10 knockouts, 10 knockdowns and 10 multifactorials. A typical reconstruction run consists of 2500 moves (1ms per move and network) on a population of 25 individual networks and takes about a minute on a single processor core. During the run, the individuals in the population usually converge to a single network with only minor variations (data not shown).

### Relative contribution of the different datasets

The contribution of the different datasets to the prediction of networks is given by dataset specific weights. The weights were derived manually based on randomly generated PNFL models. The relative contributions of the individual datasets amount to KO = 29% (w_KO_*10 data points = 80; compare eq. 7 and Table 1), KD = 21% (w_KD_*10 = 60), TC = 36% (w_TC_*100 = 100) and MF = 14% (w_MF_*10 = 40). While the combination of KO+KD accounts for half of the total dataset weights, the largest individual portion stems from the TC data.

### DREAM4 evaluation results

**Figure 7:**
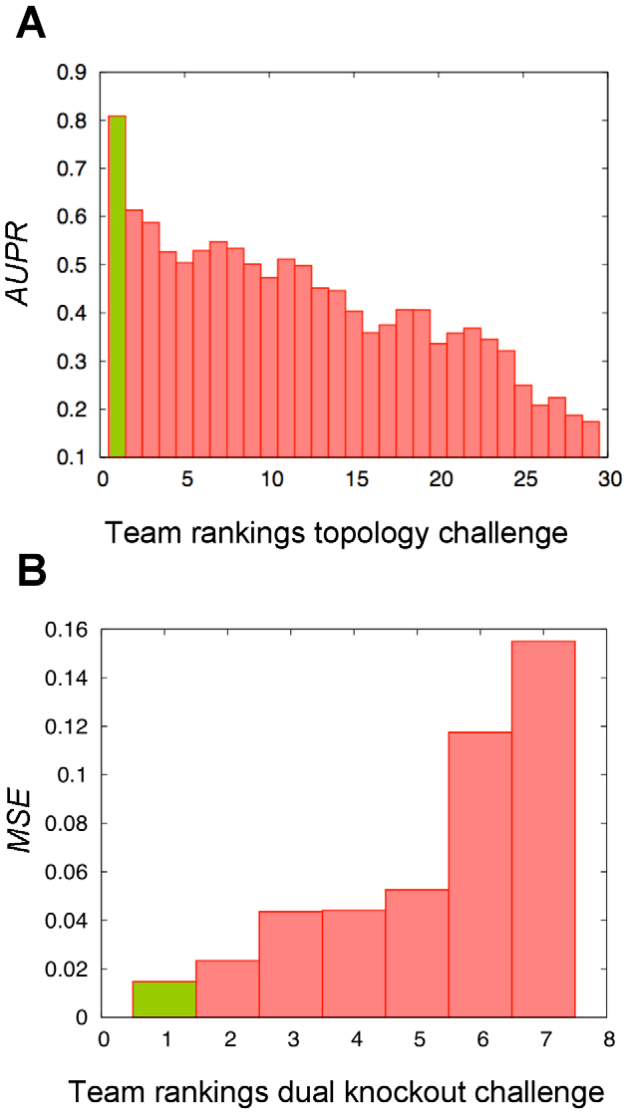
Evaluation of the *in silico* challenge comprising five networks of ten genes. Panel A shows the prediction performance of the directed unsigned topology as the area under the precision recall curve (AUPR). In a bonus challenge, steady-state level predictions of dual knockout experiments were evaluated by the mean squared error (MSE, panel B). Our performance is shown in green.

The overall results of the *in silico* size ten challenge as reported by the DREAM organizers are depicted in Fig. 7. The network topology was predicted by 29 different teams. In terms of the AUPR, our PNFL based reconstruction approach (81% AUPR averaged over 5 networks) outperformed the second best team by 20 percentage points. Our approach performed best on four of the five networks and second best on the remaining network. In an additional challenge, steady state gene levels in response to double knockout mutations were predicted. This evaluated the ability to predict the behavior of networks under previously unseen experimental conditions. Only 7 teams participated in the double knockout predictions (Fig. 7B) where PNFL also was the top performer.

### Reconstruction of network 5

**Figure 8:**
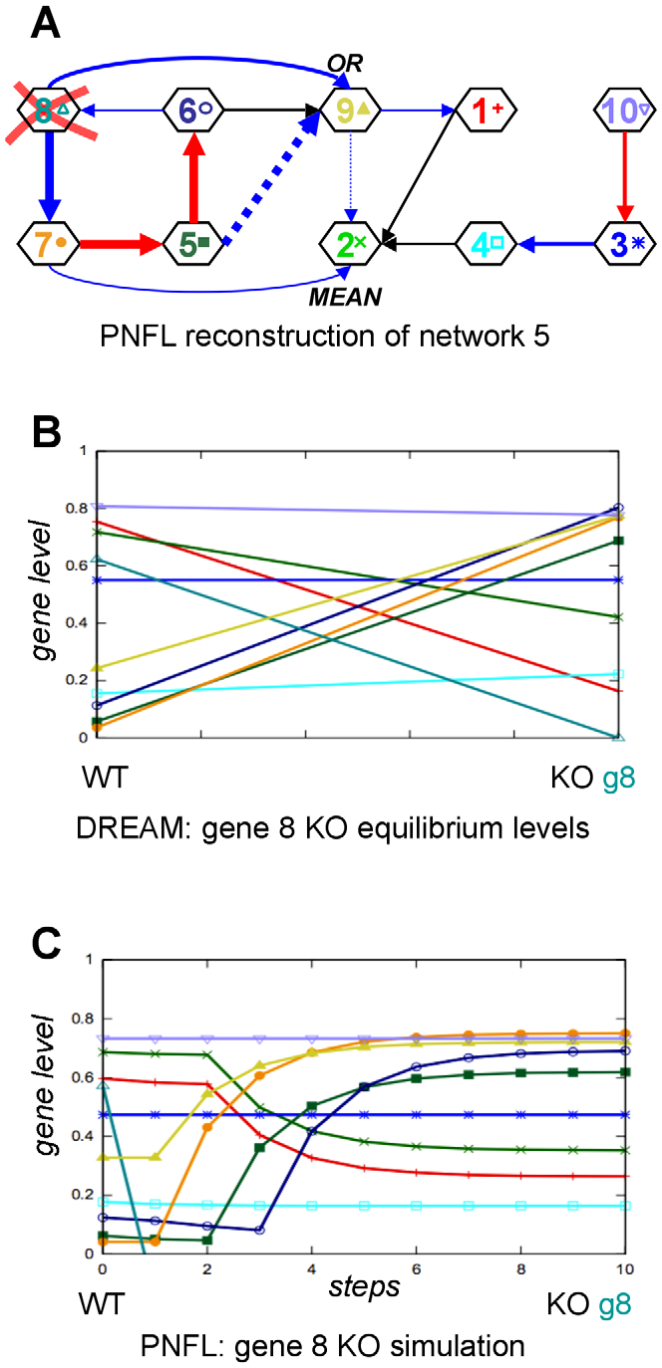
PNFL reconstruction of network 5 (AUPR = 76%). DREAM4 evaluated our predictions (panel A) in terms of correct (colored solid), missed (black) and surplus (dotted) edges. For simulation, we also infer three levels of effect strength (edge thickness) for both activation (red) and inhibition (blue). Targets regulated by multiple effectors are parametrized by the kind of regulation, i.e. dependent (*AND*, *OR*) vs. independent (*MEAN*). Incorrect predictions are more frequent when effector gene levels are low in the wild type (e.g. genes 4, 5, 6 and 9). In panels A and B we compare the provided DREAM data to the PNFL simulation for the knockout of gene 8.

Our reconstruction of network 5 (Fig. 8A) achieved an AUPR of 76%. The panels B and C in Fig. 8 compare the provided data (DREAM) to the PNFL simulation for the knockout of gene 8. Genes up (e.g. gene 7) or down regulated (e.g. gene 1) are captured correctly in the PNFL simulation. To simplify the representation of networks, transitions and the corresponding output places (compare Fig. 1) are merged into single nodes depicted as hexagons.

**Figure 9:**
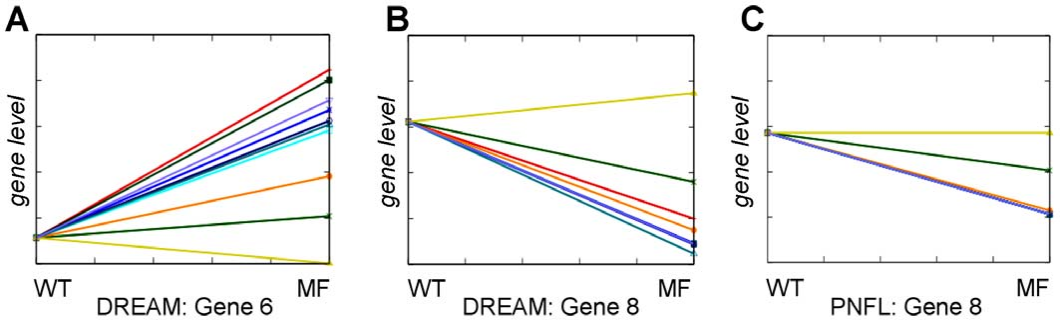
Generation of multifactorial (MF) data for an effect in network 5. In network 5, gene 6 is the only effector for gene 8 (see Fig. 8). Effectors are initialized by the provided MF gene levels (A). Subsequently, individual PNFL transitions are applied to compute the MF gene levels for the targets (C). The objective function compares the target gene levels of the provided MF data (B) to the PNFL outputs (C).

Network 5 demonstrates the utility of the multifactorial data (Fig. 9) for network reconstruction. According to personal communication at the joint RECOMB/DREAM conference 2009, several participants neglected to utilize this kind of data. The four-gene cycle (genes 5→6→8→7→5, Fig. 8A) in network 5 is an example for a difficult network motif that our approach predicts correctly only if the multifactorial data is included.

**Figure 10:**
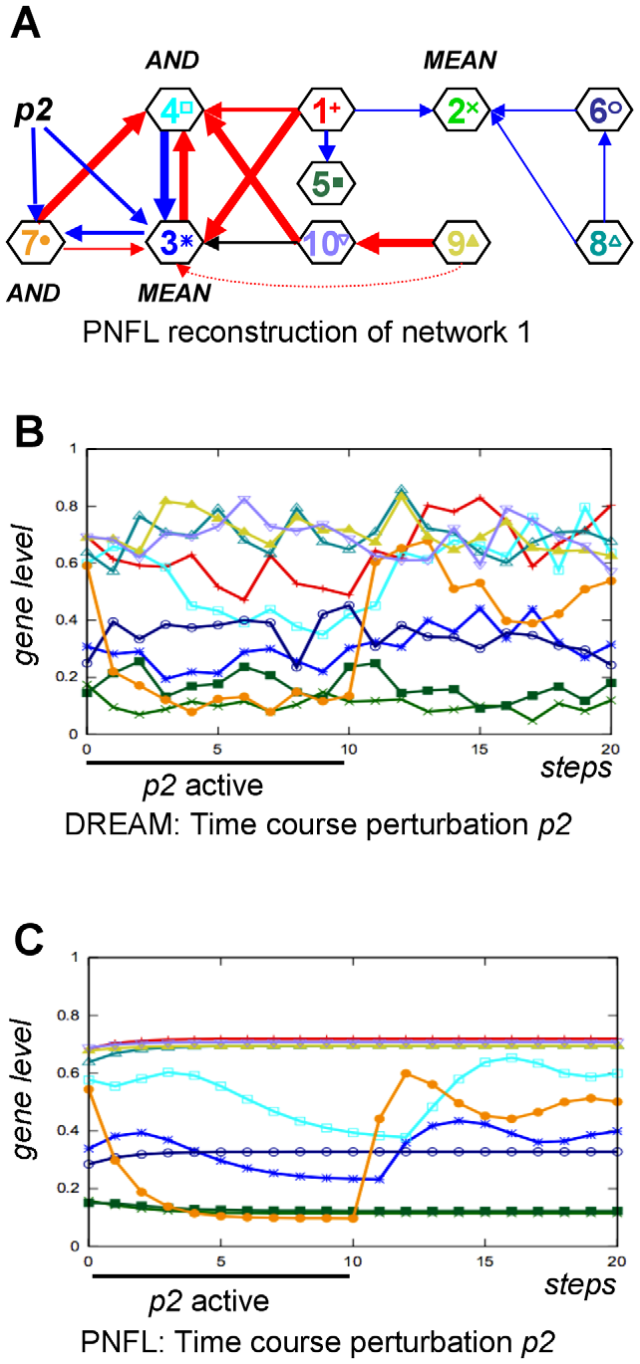
PNFL reconstruction of network 1 (AUPR = 92%). Shown is our reconstruction of network 1 (A) and the data of time course 2 as provided by DREAM (B) or simulated by PNFL (C). Time course data shows how the network responds to the application and removal of perturbations. In addition to effector targets (eq. 4), we also predict perturbation targets (eq. 6). According to our reconstruction, perturbation *p_2_* in time course 2 affects genes 3 and 7.

Incorrect predictions were more likely when the effector gene levels were low in the wild type (e.g. genes 4, 5, 6 and 9 in network 5). Here, predictions frequently contain shortcuts with respect to the true topology (Fig. 8, correct: 5→6→9, predicted 6←5→9; correct: 9→1→2, predicted: 1←9→2; see also Fig. 10, correct: 9→10→3, predicted: 10←9→3). This leads to two errors: As the effect 6→9 can not be directly observed in the given data it is missed as it is already ‘explained’ by an incorrectly predicted surplus effect (here: 5→9). Such a missing observation can for instance be due to knockouts or knockdowns exhibiting no substantial effect because of low wild type gene levels.

### Reconstruction of network 1

The reconstruction of network 1 (Fig. 10A) achieved a very high AUPR of 92%. Here, we predicted 14 out of 15 effects correctly. For a correct reproduction of time course data (e.g. time course 2 in Fig. 10, compare panels B and C) we also infer perturbation target genes. According to our reconstruction, the perturbation *p_2_* in time course 2 affects genes 3 and 7.

Network 1 was selected to demonstrate the capability of PNFL to represent oscillating network motifs. Oscillations require cycles that seem to pose no particular difficulty for the PNFL based reconstruction. Each of the three nested cycles contained in network 1 (genes 3↔4, 3↔7, 3→7→4→3) was resolved correctly. In addition, genes 3, 4 and 7 were recognized as an oscillation generating network motif. The removal of the perturbation triggers oscillations for instance in gene 7, which was picked up clearly in the PNFL simulation (Fig. 10, panels B and C).

### Validation of effect signs

The validation described in this and the following subsections is based on supplementary material posted after the completion of DREAM4 (http://gnw.sourceforge.net/resources/DREAM4%20in%20silico%20challenge.zip). It for instance enables the validation of the signs of the effects in the models, i.e. if a target is activated or inhibited by a given effector. Effect signs are determined by the effector rule tables (see Application of effector rules) selected during PNFL reconstruction. Sign predictions can only be evaluated for correct effector-target predictions. Here, the signs were predicted correctly in 100% of the cases.

### Validation of the regulatory logic

Logical operations are used by both PNFL and DREAM4 to combine the effects of multiple effectors on a given target gene. Thereby, dependent (*AND*, *OR*) and independent (*MEAN*) kinds of regulation are distinguished (see Fig. 5). In the DREAM4 setting, arbitrary combinations of these operators are possible. For instance, the activation state of gene 3 in network 1 (Fig. 5) is described by a combination of *AND* and *MEAN* operators. This is not possible by PNFL as it currently allows only one operator per target gene. Note that this does not apply to the effect signs, which are assigned to each effect separately by PNFL as well as DREAM4.

A rigorous comparison between PNFL and DREAM4 logic is not possible if more than one operator is involved as in Fig. 5. We then consider PNFL and DREAM4 regulatory logics as approximately equal if the operator that combines the majority of terms is predicted by PNFL. In the example of Fig. 5, *MEAN* (combining three terms, i.e. the three CRMs) would be correct whereas *AND* (combining two terms, i.e. TF1 and TF7) and *OR* (not used here) would be incorrect. According to this, our predictions are correct in 13 out of the 18 targets (72%) regulated by multiple effectors. Three of the five mismatches are explained by topological errors. Here, the corresponding target genes are connected to single effectors in the PNFL models and to multiple effectors in the DREAM4 network. Thus, if the predicted topology permits the inference of the regulatory logic it is correct in 87% ( = 13/15) of the cases.

### Validation of time course perturbation targets

The time courses emerge from perturbations that affect a specific subset of target genes. The PNFL based simulation of the time courses thus required the prediction of the targets of a perturbation (see Time course data). The evaluation of our prediction performance on inferring the time course perturbation targets resulted in an AUPR of 73%. The performance difference to the prediction of effector targets (AUPR of 81%) is due to the fact that each perturbation corresponds to a single time course. The remaining four time courses (and all of the KO, KD and MF datasets) do not provide any information with regard to the targets of a selected perturbation.

## Discussion

We presented a method for network reconstruction that uses Petri Nets with Fuzzy Logic (PNFL) for modeling and simulation. This approach was the best performer (Fig. 7) in the *in silico* size ten challenge of the 2009 DREAM4 assessment of reverse engineering methods. Why did it work so well?

Our approach optimizes models to achieve the best possible agreement between PNFL generated datasets and the datasets provided in the DREAM4 challenges. To get the most out of the data, we employ specific simulation approaches for each of the available datasets. This allows us to exploit and score heterogeneous datasets in a unified way. We further reduce the model complexity severely to avoid the risk of overfitting. Ideally, only a single network should be able to reproduce the data. The model space is still huge, requiring a heuristic, population based search strategy. It avoids local minima traps and thus improves the convergence of networks and also the agreement between PNFL and DREAM datasets. The resulting PNFL models accurately predict the network behavior even under new experimental conditions not seen during model building. This was demonstrated in the double knockout challenge (Fig. 7B).

Incorrect predictions might result when the effector gene levels are low in the wild type. Knockout experiments, for instance, provide only little topological information in such cases. This is particularly frequent in network 2 (data not shown) where different network topologies generate similar data and our reconstruction does not converge to a single network. Indeed, no team achieved a good prediction performance for network 2. In case of genes with low wild type expression, over-expression instead of knockout experiments should be performed.

Several of the participating teams focused on the knockout (KO) datasets and neglected to exploit the time course (TC), multifactorial (MF) and knockdown (KD) data in their reconstruction (personal communication at the joint RECOMB/DREAM conference 2009). We found that only the combination of all provided datasets enabled us to predict particularly difficult network motifs. An example is the unusual four-node cycle in Fig. 8A that is predicted correctly only when using the MF data (Fig. 9). In general, cycles and nested cycles pose no particular difficulty to our PNFL based approach (e.g. Fig. 10A). The time course shown in Fig. 10 demonstrates that our reconstruction also resolves and recognizes oscillating network motifs.

The reconstruction of PNFL models reliably determines a range of mechanistic details that go beyond the graph topology evaluated in DREAM. Our models distinguish activation from inhibition, dependent from independent regulation as well as strong, medium and weak degrees of effect strength. Such intuitive assertions are sufficient to specify, visualize and thus comprehend executable models and their parameters. This is a characteristic feature of fuzzy logic modeling [18]. Similarly intuitive notions are more difficult if not impossible to obtain from ODE, mutual information or Bayesian models. Nevertheless, both PNFL and ODE enable the detailed simulation of models. Simulation models can facilitate an iterative cycle of model improvements based on the comparison between *in silico* and laboratory experiments.
